# 1,5-Anhydro-D-Mannitol Is a Potentially Low-Energy Carbohydrate: A Dual-Model Approach in Rats and a Randomized, Three-Way Crossover Trial in Humans

**DOI:** 10.3390/nu18111825

**Published:** 2026-06-05

**Authors:** Kenichi Tanabe, Ikuma Tanaka, Hiromi Hayashi, Kazuhiro Yoshinaga, Sadako Nakamura

**Affiliations:** 1 Department of Nutritional Science, Faculty of Nutritional Sciences, Nakamura Gakuen University, 5-7-1 Befu, Jonan-Ku, Fukuoka 814-0198, Japan; 2 Graduate School of Nutritional Science, Nakamura Gakuen University, 5-7-1 Befu, Jonan-Ku, Fukuoka 814-0198, Japan; 3 SUNUS Co., Ltd., 20 Nanei 3-Chome, Kagoshima City 891-0196, Japankazu-yoshinaga@sunus.co.jp (K.Y.); 4 Graduate School of Human Life Sciences, Jumonji University, 2-1-28 Sugasawa, Niiza 352-8510, Japan; nacsacpv@gmail.com

**Keywords:** 1,5-anhydro-D-mannitol, bioavailability, breath hydrogen gas, urinary excretion, crossover study, gut fermentation

## Abstract

Background/Objectives: We hypothesized that orally ingested 1,5-anhydro-D-mannitol (AM) would be absorbed from the gut but poorly metabolized, and it may have low fermentability by gut microbiota based on the breath hydrogen test. Methods: We used a dual-model approach in this study. In animal experiments, AM was administered orally to male Wistar rats. In a randomized, three-way crossover trial (UMIN000054040), 15 healthy young adults consumed a single 5 g dose of AM, mannose, or fructooligosaccharide (FOS). Primary outcome was breath hydrogen concentrations; secondary outcomes included plasma AM, blood glucose concentration, and urinary AM excretion. Results: After ingestion of 5 g AM in humans, the 14 h area under the curve for breath hydrogen excretion was markedly lower after AM than that after FOS ingestion (*p* < 0.05). Plasma AM peaked at 1 h, whereas blood glucose remained unchanged from baseline. Approximately 30% of ingested AM was excreted unchanged in urine within 24 h. Results of animal experiments were similar to those in humans. Fecal excretion in AM was confirmed to be minimal, accounting for ~1% of the administered dose within 24 h in rats. Conclusions: These findings indicate that AM is potentially a low-energy carbohydrate.

## 1. Introduction

1,5-anhydro-D-mannitol (AM), also known as 1-deoxy-D-mannose, is the C1-deoxy form of D-mannose (Man) [1]. AM can be synthesized by hydrogenating 1,5-anhydro-D-fructose (1,5-AF), which is produced from α-glucan using palladium/carbon (Pd/C) as a catalyst. Alternatively, *Saccharomyces cerevisiae* can enzymatically reduce 1,5-AF in culture, releasing AM together with 1,5-anhydro-D-glucitol, the C2 epimer of AM [2,3]. A previous study has shown that orally ingested AM is excreted in the human urinary tract without undergoing metabolism [3]. However, human trials of oral AM ingestion (1 g) reported urinary excretion rates of approximately 40% within 8 h [3]. These findings suggest that unabsorbed AM in the small intestine may reach the large intestine, where it could be fermented by gut microbiota; if not fermented, it is likely excreted in feces.

One of the methods for measuring the energy value of test substance involves the classical approach of collecting feces and urine over a long-term following the ingestion of the test substance and calculating the energy value [4]. We evaluated the bioavailability and available energy of various nondigestible or nonabsorbable saccharides, including 1,5-AG, in rat and human studies [5,6]. These evaluations were conducted by measuring breath hydrogen gas as an indirect primary indicator of gut fermentability, alongside comprehensive assessments of digestibility, absorbability, and the rates of urinary and fecal excretion. Other methods for ascertaining the available energy values of these saccharides in human studies have been reported, including a combination of indirect calorimetry and breath hydrogen measurement [7] as well as methods that allow for the indirect assessment of energy content through the continuous intravenous infusion of 13C-labeled short-chain fatty acids [8]. However, there are currently no internationally standardized methods for evaluating the bioavailability and the available energy of these saccharides. The evaluation method used in this study has been established as a method authorized by the Japan Society of Dietary Fiber Research (so called “JDF method”) [9]. Furthermore, in Japan, according to the report provided by the Consumer Affairs Agency, Government of Japan [10], the energy conversion factors for these saccharides must be strictly determined based on in vivo experimental data, specifically evaluating gut fermentability using breath hydrogen excretion as the primary indicator, supported by secondary indices including blood parameters, and urinary and fecal excretion rates. Consequently, in Japan, other researchers also use this method to evaluate the available energy content of these saccharides [11,12]. For example, we demonstrated that 1,5-AG, a metabolite of 1,5-AF, is almost completely absorbed in the small intestine and excreted in urine following oral ingestion [5]. Because of this efficient absorption, it is not fermented by gut microbiota. Conversely, Kitagawa et al. used experimental methods similar to ours to investigate the bioavailability of mannose [11]. They reported that more than 90% of orally ingested mannose escapes absorption in the small intestine and is fermented by gut microbiota in the large intestine. Since absorbed mannose is not excreted in urine, it is likely metabolized to glucose and utilized as an energy source.

Considering the established in vivo dynamics of 1,5-AG and mannose, we can anticipate the behavior of AM after oral ingestion. AM is expected to be absorbed efficiently. However, its unique C-1 configuration prevents phosphorylation by hexokinase, the first irreversible step in glycolysis, leaving it metabolically inert. Consequently, AM would likely have minimal effects on blood glucose, and a substantial fraction of the absorbed compound should be rapidly excreted in urine unchanged. This profile directly addresses the bioavailability limitations of AM. Furthermore, given the low fermentability of the structurally similar 1,5-anhydro-D-glucitol [5], we hypothesized that any unabsorbed AM reaching the colon would be only minimally fermented by gut microbiota.

To test this central hypothesis, we designed a dual-model approach which was the present study to comprehensively quantify the in vivo dynamics of AM in rats and healthy young adults. Our previous studies have evaluated the bioavailability of a newly developed saccharide in Wistar rats and humans, yielding similar results [5,6]. In animal experiments, AM was administered orally to male Wistar rats to verify the validity of the results in the experiment. Primary outcome was extracorporeal hydrogen concentration as a marker of colonic fermentation. Secondary outcomes included plasma AM concentration and 24 h fecal and urinary AM excretion directly assessing its absorption and elimination. The human experiment used a rigorous, randomized crossover approach. The primary outcome was breath hydrogen concentration. Secondary outcomes included plasma AM concentration, blood glucose levels to gauge glycemic impact and 24 h urinary AM excretion. For comparison, we included mannose and fructooligosaccharide (FOS), a well-established nondigestible oligosaccharide as the reference substance defined by the JDF method. Clarifying these physiological characteristics is essential for determining its available energy.

This work is expected to advance the field by providing more detailed fundamental data on AM bioavailability in rats and humans, and delivering key scientific evidence for its distinctive physiological effects.

## 2. Methods and Materials

### 2.1. Animal Experiments

#### 2.1.1. Experimental Animals and Ethical Considerations

We acquired 8-week-old male Wistar rats (~220 g body weight) from CLEA Japan, Inc. (Tokyo, Japan). Animals were housed individually under controlled conditions at 22 °C ± 1 °C, 50% ± 10% humidity, and a 12 h light/dark cycle (lights on 7:00 A.M.–7:00 P.M.). Before the experiment, all rats had *ad libitum* access to a standard purified diet based on the AIN-93M formulation and water for 5 days. Rats were randomly assigned to four groups. A random allocation sequence was generated using a computer’s random number function by Sadako Nakamura. All procedures were approved by the Animal Research Ethics Committee of Jumonji University (approval number: 2309) and conducted in accordance with the Guide for the Care and Use of Laboratory Animals (National Research Council, MD, USA) and the Standards Relating to the Care and Management of Experimental Animals (Notification No. 88, Prime Minister’s Office, Tokyo, Japan). All experiments were performed at the Laboratory of Health and Nutritional Sciences, Jumonji University.

#### 2.1.2. Test Substances and Administration

AM was synthesized by the method reported by Andersen et al., with some modifications as previously reported [13]. The purity of the resulting AM was 98.2%, as determined by high-performance liquid chromatography using an MCI GEL CK 08 S column (i.d. 0.8 cm × 60 cm; Mitsubishi Chemical Corp., Tokyo, Japan.) with water as the eluent at a flow rate of 1 mL/min and 60 °C. Eluted materials were detected using a differential refractometer (Jasco Corp., Tokyo, Japan).

A 14-day single-dose oral AM administration caused no death or systemic abnormalities and necropsy revealed no abnormalities in mice. Based on these results, the LD50 value of AM in female mice is >2000 mg/kg [3]. FOS is almost entirely utilized by intestinal bacteria after reaching the large intestine without digestion in the small intestine [5]. Thus, FOS (purity > 98%; Meiji Co., Ltd., Tokyo, Japan) served as positive fermentability control and erythritol as a negative control, easily absorbed and excreted into urine, and not much fermented by gut microbiota (ERT; purity > 99%; Bussan Food Science Co., Ltd., Aichi, Japan).

Administration: In the single oral administration experiment, rats were assigned to four groups after a 15 h fast: AM 500 mg/kg body weight (n = 6), AM 1000 mg/kg body weight (n = 6), FOS 400 mg/rat (n = 6), and ERT (n = 3). A total of 21 rats were used in this study. The sample size and dosages were determined based on our previous studies evaluating the bioavailability of novel saccharides in rats [5]. Specifically, the AM dose was based on a previous safety test in mice [3]. Meanwhile, the FOS dose was selected to allow direct comparison with our previous data and avoid inducing hyperosmotic diarrhea [5]. All substances were administered by gastric tube. The animals were sacrificed under anesthesia using isoflurane inhalation (concentration 2–3%; flow rate 1.4 L/min) before collecting samples. Gastric tube administration was performed by trained personnel using appropriately sized equipment, following standard procedures to minimize animal distress. During and after administration, no expected or unexpected adverse events were observed. Consequently, no specific interventions to reduce pain or suffering were required, and no humane endpoints were established.

#### 2.1.3. Sample Collection

Extracorporeal hydrogen gas: Following AM, FOS, or ERT administration, each rat was placed in an individual circulatory metabolic measurement apparatus (Sugiyamagen-Iriki Co., Ltd., Tokyo, Japan). Measurements began at approximately 9:00 AM with airflow maintained at 200 ± 30 mL/min. After a 30 min acclimation period, a 40 mL circulating air sample was collected as the 0 min measurement. Subsequent air samples were collected every hour for up to 10 h.

Blood: Blood samples were obtained from the tail vein at 0, 1, 2, 3, 4, 5, and 6 h after single AM administration. Plasma was separated and stored at −80 °C until analysis.

Urine: Urine samples for AM measurement were collected before dosing and at 10 and 24 h post-administration.

Feces: Fecal samples were collected over 24 h in metabolic cages after single AM administration, weighed, and stored at −80 °C until analysis.

#### 2.1.4. Analytical Methods

The animal experiment was open-label. However, for analyses outsourced to SUNUS Co., Ltd., (Kagoshima, Japan) samples were coded to blind the analysts to group allocation.

Extracorporeal hydrogen gas analysis: Extracorporeal hydrogen gas concentration was determined with a gas chromatograph–based H_2_/CH_4_ Breath Gas Analyzer BGA-1000D (Laboratory for Expiration Biochemistry Nourishment Metabolism, Nara, Japan).

Blood, urine, and feces analysis: To 30 μL of undiluted plasma, urine, or feces (undiluted, 5-fold diluted, or 10-fold diluted), 60 μL of ethanol and 0.45 μg of adonitol as an internal standard were added, and the mixture was centrifuged at 4 °C and 20,000× *g* for 5 min. The supernatant was then dried in a centrifugal evaporator at 70 °C. After drying, 20 μL of TMS-PZ (Tokyo Chemical Industry Co., Ltd., Tokyo, Japan.) was added to the dried sample, which was sonicated for 5 min and incubated at room temperature for 1 h for TMS derivatization.

GC-MS analysis: A gas chromatograph–mass spectrometer (GC: TRACE 1610; MS: TSQ 9610; Thermo Fisher Scientific Ink., Waltham, MA, USA) was used with a TG-5SILMS column (20 m length, 0.18 mm I.D., 0.18 μm film). Conditions: temperature program 80 °C to 300 °C at 20 °C/min; injector temperature 250 °C; split ratio 20:1; helium carrier gas (0.8 mL/min); injection volume 1 μL; ion source temperature 250 °C; scan range *m*/*z* 50–550. Fragment ions at *m*/*z* 217 for AM, and *m*/*z* 204 for mannose were extracted for peak area calculation. Concentrations of were quantified using the peak area at *m*/*z* 204 for alditol.

### 2.2. Human Experiments

#### 2.2.1. Ethical Approval

This study complied with the Declaration of Helsinki, and all procedures involving human participants were approved by the ethics committee of Nakamura Gakuen University (approval number: 2023-021). The trial was registered with UMIN-CTR (ID: UMIN000054040). The full protocol is not publicly accessible. It can be obtained from the corresponding author upon reasonable request.

#### 2.2.2. Study Design

This study used a randomized, three-way crossover design based on our previous work [5,6]. Participants ingested the test substances (5 g AM, 5 g FOS, or 5 g mannose) in random order, with an allocation ratio of 1:1:1 for each sequence.

**Figure 1:**
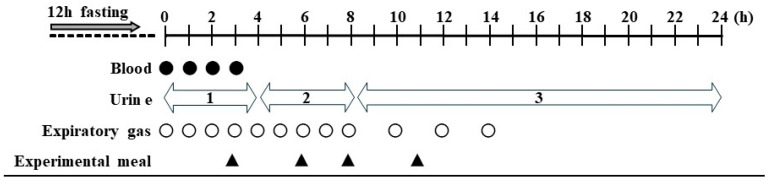
Schematic of the study protocol.

The sequence was generated by an investigator using a computer’s random number function. The trial was conducted in an open-label format, as blinding of participants and investigators was not feasible due to differences in assessment protocols, such as urine collection duration, between test substances. Consequently, allocation concealment was not applied. The ingestion protocol is illustrated in Figure 1. A washout period of at least 1 week separated each trial period to eliminate carryover effects on the intestinal lumen. No substantive changes to study methods occurred after initiation. For reference, AM 1 g was also evaluated in an exploratory manner using the identical protocol in a subset of participants (n = 6, randomly selected from those who ingested AM 5 g). The trial did not mandate or restrict any specific concomitant care or interventions.

This randomized, single-dose, three-way crossover trial in healthy adults involved ingestion of 5 g AM, 5 g mannose, or 5 g fructooligosaccharide (FOS) in random order. A washout period of at least 1 week separated each intervention. On each test day, after an overnight fast, baseline blood, urine, and breath samples were collected prior to ingestion of the test substance, followed by repeated sampling over 14–24 h.

#### 2.2.3. Participants

Healthy adult men and women (aged 20–29 years) from Nakamura Gakuen University were recruited. All participants received a full explanation of the study’s purpose, procedures, and anticipated benefits and risks before providing written informed consent. Exclusion criteria included a history of diabetes, glucose metabolism disorders, respiratory disease, chronic gastrointestinal conditions (e.g., inflammatory bowel disease, irritable bowel syndrome, functional dyspepsia), abdominal surgery, abnormal bowel habits, blood disorders, food allergies, or a BMI of ≥25.0. Smokers, individuals with excessive alcohol intake, and pregnant or lactating women were also excluded. Those who regularly consumed nondigestible saccharides (e.g., dietary fiber) or routinely used medications, foods for specified health uses, foods with function claims, or other health foods likely to affect study outcomes were likewise excluded. Baseline participant characteristics are summarized in Table 1.

No patients or members of the public were involved in designing, conducting, or reporting this research. This study was conducted at the Health Promotion Center of Nakamura Gakuen University in Fukuoka, Japan, between June and September 2024.

Values are mean ± standard deviation (SD) for 15 participants (3 men, 12 women). BMI, body mass index.

#### 2.2.4. Experimental Protocol and Assessments

The human study was conducted in accordance with JDF method [5,6].

Test substances: AM, fructooligosaccharide (FOS, positive control), and mannose (purity > 98%; NOW Foods, USA) as a comparative control were used.

Outcomes: The primary outcome was breath hydrogen output. Secondary outcomes were plasma AM, blood glucose concentration and urinary AM excretion.

Procedures: Detailed methods are described in the respective sections below (Figure 1). For mannose, urine collection was limited to 8 h because it is known to be minimally excreted in urine [11]. For FOS, only breath hydrogen measurements were collected. Each test substance was dissolved in lukewarm water and consumed by participants while fasting under the direct supervision of the research staff. As a result, adherence was 100% for all completed sessions, and all interventions were delivered as intended with no protocol deviations.

Safety assessment: Safety was monitored throughout the study. All adverse events, including gastrointestinal symptoms (e.g., diarrhea, abdominal bloating) and issues related to self-blood collection from the fingertip, were recorded. Participants were instructed to immediately report any adverse events to the study staff. Before each intervention, staff conducted a health check that included blood pressure and pulse measurements and a brief interview. The fingertip condition was examined after each test and again the following day. All blood collection procedures were performed at Nakamura Gakuen University’s Health Promotion Center, where a physician or nurse was available to provide immediate medical care if required.

#### 2.2.5. Analytical Methods

The analytical methods for breath hydrogen gas concentration and for plasma and urinary AM concentrations were as described in the above protocol, with minor modifications for human samples. Blood glucose was measured by the Trinder method, which uses glucose oxidase [14].

#### 2.2.6. Sample Size Determination

This investigation was exploratory in nature. The sample size of 15 participants was selected based on prior research on breath hydrogen excretion (sample sizes of 10–25) and feasibility constraints [5,6].

### 2.3. Statistical Analyses

Results are presented as mean ± standard deviation (SD). In the animal experiment, after verification of a normal distribution was verified, and group comparisons were conducted using analysis of variance (ANOVA) followed by Tukey’s post hoc test. A *p*-value < 0.05 was considered statistically significant. The area under the curve (AUC) for extracorporeal hydrogen gas concentration was calculated by the trapezoidal rule. In human experiment, descriptive statistics were calculated for the area under the curve (AUC) for breath hydrogen excretion and plasma AM concentration. After verifying a normal distribution, a repeated measures ANOVA (RM-ANOVA) with Bonferroni’s post hoc test was used to compare parameters across test substances. The validity of breath gas excretion levels as an indicator of fermentability was evaluated using a one-sample *t*-test. Statistical analysis was performed using IBM SPSS Statistics ver. 25 (IBM Corp., Armonk, NY, USA), with significance set at *p* < 0.05 (two-tailed). Data for the AM 1 g group (n = 6) were considered exploratory and limited to descriptive statistics.

All results obtained from animal experiments were included. Therefore, no exclusion criteria were established.

An interim analysis was neither planned nor conducted, and the pre-specified stopping criteria were not applied. Subgroup and sensitivity analyses were also not planned or performed, and no additional post hoc analyses were undertaken. The principal investigator had planned to discontinue participation for any subjects if continued involvement was considered unsafe due to adverse events or other health concerns; however, no such cases occurred.

## 3. Results

### 3.1. Animal Experiments

#### 3.1.1. General Condition, Body Weight Changes, and Diarrhea

During the experimental period, no abnormalities in body condition or weight loss were observed in any animals. No osmotic diarrhea or soft stools were observed after single oral AM administration.

#### 3.1.2. Extracorporeal Hydrogen Gas Excretion

**Figure 2:**
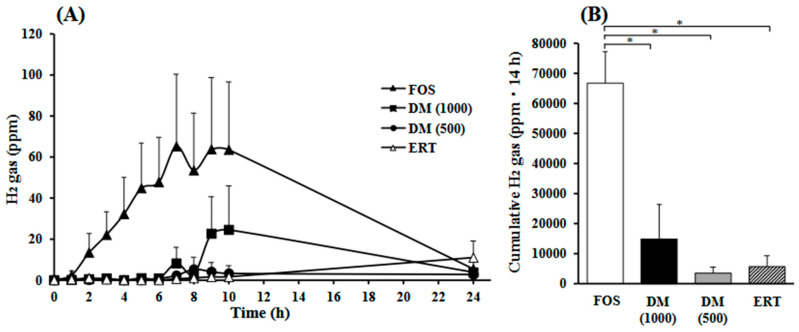
Gut fermentability of AM after a single oral administration in male Wistar rats. (**A**) Extracorporeal hydrogen gas excretion over time after AM administration (500 mg/kg, ●; 1000 mg/kg, ■), positive control: fructooligosaccharide (FOS, ▲), or negative control: erythritol (ERT, △). (**B**) Area under the curve (AUC) for breath hydrogen excretion over 10 h. Values are mean ± SD (n = 6 for AM and FOS groups; n = 3 for ERT group). * Significantly different from the FOS group (*p* < 0.05), analyzed by ANOVA with Tukey’s post hoc test.

The time course of hydrogen gas concentration is shown in Figure 2A. The FOS group (positive control) exhibited marked extracorporeal hydrogen gas excretion. In AM groups (500 and 1000 mg/kg), excretion levels tended to be higher than in the ERT group (negative control) but still extremely low compared with the FOS group. The 24 h AUC for extracorporeal hydrogen gas excretion was significantly lower in both the AM and ERT groups compared with the FOS group (*p* < 0.05; Figure 2B).

#### 3.1.3. Plasma AM Concentration

**Figure 3:**
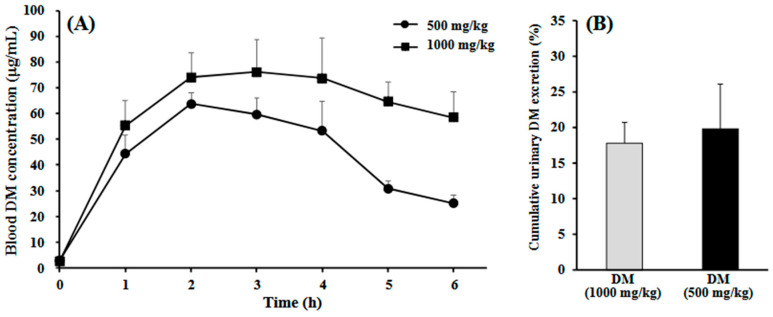
Bioavailability of 1,5-anhydro-D-mannitol (AM) after a single oral administration in male Wistar rats. (**A**) Plasma AM concentrations after a single oral dose of 500 mg/kg (●, n = 6) or 1000 mg/kg (■, n = 6). (**B**) Cumulative urinary AM excretion (% of ingested dose over 24 h following a single oral dose). Values are mean ± standard deviation (SD).

After a single oral AM dose, plasma AM concentration peaked at 2 or 3 h, before gradually declining (Figure 3A). Peak concentrations were 74.2 ± 9.5 μg/mL in the 1000 mg/kg group and 63.8 ± 4.3 μg/mL in the 500 mg/kg group.

#### 3.1.4. Urinary AM Excretion

The cumulative urinary AM excretion after a single oral dose increased in a dose-dependent manner (Figure 3B). At 24 h, cumulative excretion was 33.9 ± 6.2 mg (17.8 ± 2.9% of dose) in the 1000 mg/kg group and 22.9 ± 7.9 mg (19.8 ± 6.3% of dose) in the 500 mg/kg group.

#### 3.1.5. Fecal AM Excretion

Fecal AM excretion during the 24 h after a single oral dose was 1.2 ± 1.6 mg (0.7 ± 1.0% of dose) in the 1000 mg/kg group and 0.5 ± 0.6 mg (0.3 ± 0.4% of dose) in the 500 mg/kg group.

### 3.2. Human Experiments

#### 3.2.1. Participant Flow and Background

**Figure 4:**
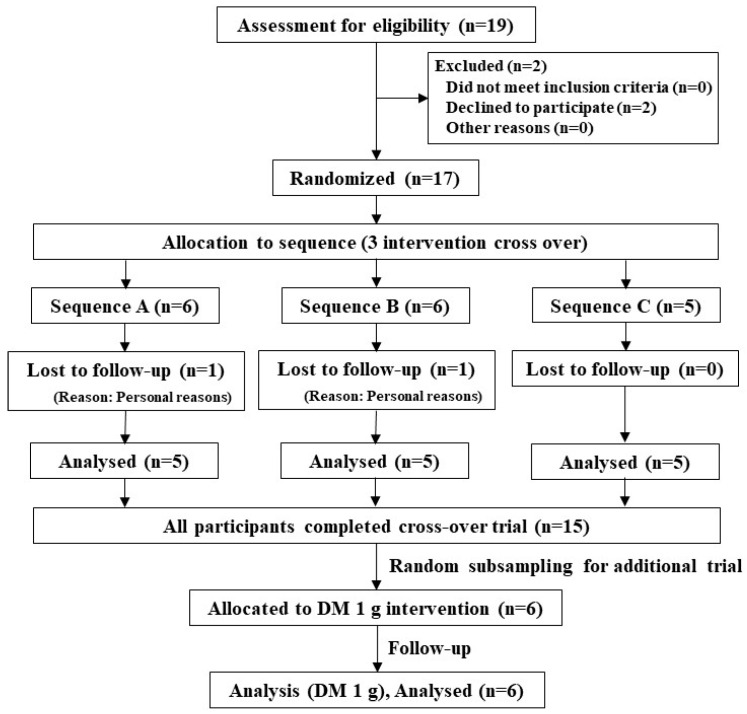
CONSORT 2025 flow diagram of the study.

Participants were recruited in April–May 2024, and the trial was conducted from June 2024 to September 2024. The participant flow is illustrated in Figure 4. Seventeen individuals met the eligibility criteria, provided consent, and were randomized. Fifteen completed all trial periods and were included in the primary outcome analysis. Two participants withdrew for personal reasons unrelated to adverse events from the test substances. The final analysis included 15 participants (mean ± SD: age 21.8 ± 1.1 years, height 160.4 ± 5.5 cm, weight 54.1 ± 6.7 kg, BMI 21.0 ± 2.1 kg/m^2^). No serious adverse events occurred during any trial period.

The diagram illustrates participant flow through enrollment, allocation to intervention sequences, follow-up, and inclusion in the analysis.

#### 3.2.2. Breath Hydrogen Excretion

**Figure 5:**
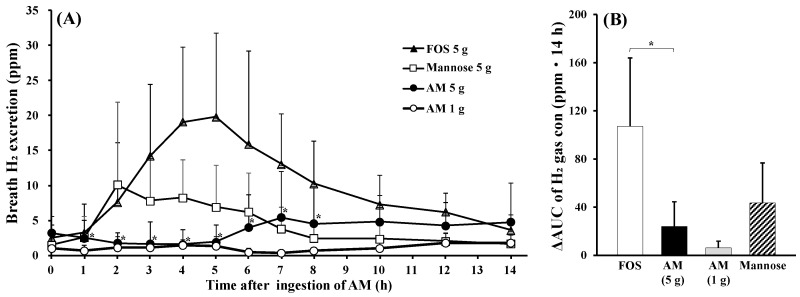
Breath hydrogen (H_2_) excretion (**A**) and ΔAUC0-14 h (**B**) after a single oral ingestion of test substances in healthy adults. Time course of breath H_2_ concentration from baseline after ingestion of 5 g AM (●, n = 15), 1 g AM (○, n = 6), 5 g mannose (□, n = 15), or 5 g fructooligosaccharide (FOS; ▲, n = 15). The AUC (ΔAUC0-14 h) for breath H_2_ excretion was calculated from baseline-corrected concentrations over 14 h. Values are mean ± SD. * Significantly different from the FOS group (*p* < 0.05) compared with the 5 g AM group, analyzed by repeated measures ANOVA with Bonferroni’s post hoc test.

After FOS ingestion, breath hydrogen concentration rose until 5 h (20.0 ± 12.0 ppm) and then gradually declined (Figure 5A). After AM ingestion, there was no significant change from baseline, with only a slight increase after 6 h (4.0 ± 4.7 ppm). Following mannose ingestion, breath hydrogen excretion rose rapidly, peaked at 2 h (10.1 ± 11.8 ppm), and then gradually decreased. The ΔAUC_0–14h_ was significantly lower for the 5 g AM group (24 ± 21 ppm·14 h) than for the FOS group (107 ± 57 ppm·14 h; *p* < 0.05) (Figure 5B). Relative to FOS (100%), the ΔAUC_0–14 h_ for AM and mannose was 22% and 41%, respectively.

#### 3.2.3. Changes in Plasma AM Concentration

**Figure 6:**
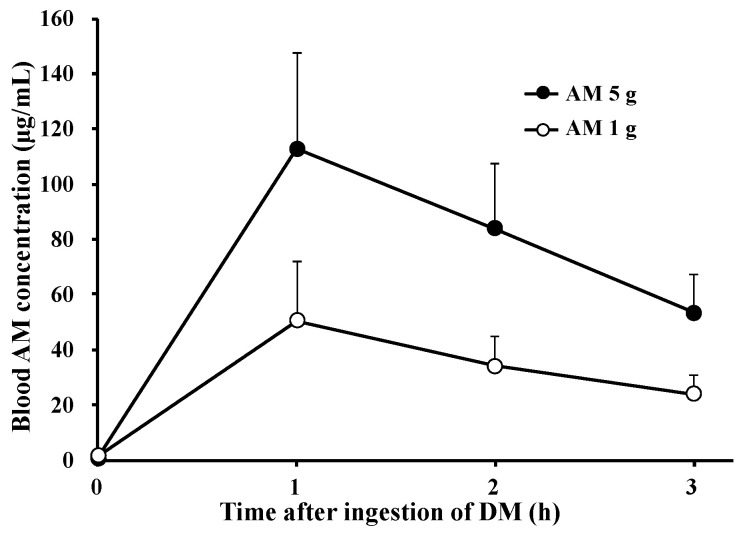
Changes in plasma 1,5-anhydro-D-mannitol (AM) concentrations in healthy adults. Time course of plasma concentrations after a single oral ingestion of 5 g AM (●, n = 15) or 1 g AM (○, n = 6; reference). Plasma AM peaked at 1 h post-ingestion. Values are mean ± SD.

Following ingestion of 5 g AM, plasma AM concentration peaked at 1 h (113 ± 35 µg/mL) and gradually declined to 53 ± 14 µg/mL by 3 h (Figure 6).

#### 3.2.4. Urinary AM Excretion

AM was detected in urine, with a cumulative 24 h excretion of 1.5 ± 0.3 g after a 5 g dose, corresponding to a urinary excretion rate of 29.5 ± 6.6% of the ingested amount (Table 2). In contrast, urinary excretion of mannose remained extremely low (nearly 0%) over 8 h.

#### 3.2.5. Changes in Blood Glucose Concentration

**Figure 7:**
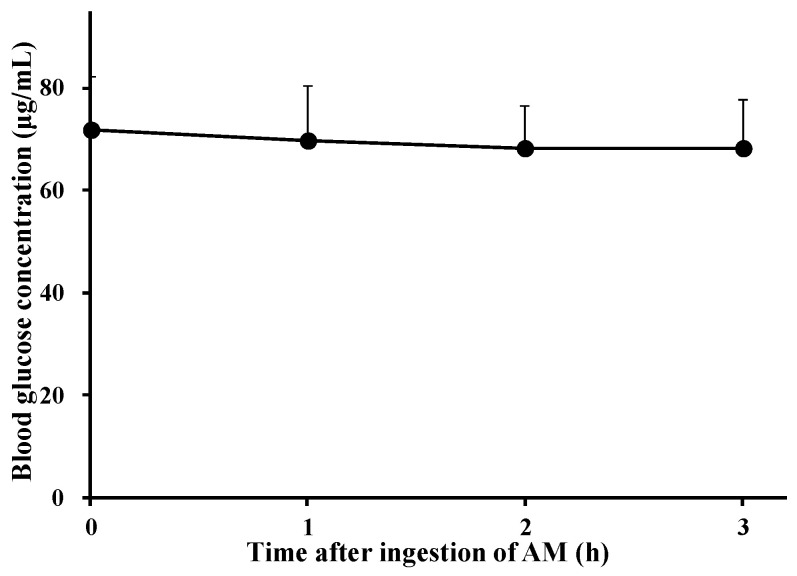
Changes in blood glucose concentrations in healthy adults. Blood glucose showed no significant change from baseline after ingestion of 5 g AM (●, n = 15). Values are mean ± SD.

After ingestion of 5 g AM, no statistically significant changes in blood glucose from baseline were observed (Figure 7).

## 4. Discussion

As hypothesized, this study demonstrates that AM is a non-glycemic saccharide with a distinctive fermentation profile. In human experiment, our primary findings show that orally ingested AM is rapidly absorbed in the small intestine, does not alter blood glucose levels, is partially excreted unchanged in urine, and produces significantly less hydrogen gas than FOS. This animal experiment also showed that orally administered AM in male Wistar rats is rapidly and partially absorbed into the bloodstream, with an unchanged part in urine. Extracorporeal hydrogen gas excretion was markedly lower than that of FOS, a typical nondigestible oligosaccharide, indicating that AM is poorly fermented by Wistar rat gut microbiota. The minimal fecal excretion within 24 h, accounted for <1% of the administered dose. These results align with human study findings that AM is absorbed from the gastrointestinal tract without affecting blood glucose and exhibits low intestinal fermentability. Notably, the minimal fecal excretion provides key evidence for evaluating AM’s energy value. Overall, these findings confirm that AM is a novel, low-fermentability functional carbohydrate.

The observation that plasma AM concentration peaked 1 h after a 5 g dose indicates efficient small-intestinal absorption in human experiment. However, its bioavailability pattern differs markedly from common saccharides, as no significant changes in blood glucose were detected. This suggests AM is unlikely to serve as an energy source. Although hexokinase can phosphorylate 1,5-AG—compounds structurally similar to AM [15]—its unique C-1 deoxy configuration likely prevents further metabolism through glycolysis. Studies have shown that deoxy sugars, even when phosphorylated by hexokinase, cannot be used as an energy source because they are not metabolized in the glycolytic pathway [16]. As a result, absorbed AM does not enter major saccharide metabolic pools, and roughly 30% of the ingested dose is excreted unchanged in urine within 24 h. Its excretion profile resembles that of other low-metabolizable saccharides, such as erythritol (~90% urinary excretion) [17] and D-allulose (~70% urinary excretion) [18].

It is assumed that roughly 70% of ingested AM reaches the large intestine without being absorbed, but our findings suggest this is unlikely. The AUC for breath hydrogen after a 5 g AM dose was very low—about 22% of that for FOS, the positive control known to be almost completely fermented. The FOS AUC in our study (107 ppm·14 h) aligns with previous reports, supporting the validity of our measurement system [6]. Previous study has evaluated the ability of *E. coli* (NBRC 3301) to utilize AM [3]. No increase in turbidity was observed in the AM-containing culture solution compared to the culture solution containing mannose or glucose. These results indicate that unabsorbed AM is poorly metabolized by gut microbiota, and its contribution to host energy via short-chain fatty acids is minimal. Notably, mannose, a structural analog, showed greater fermentability than AM (AUC of 44 ± 33 ppm·14 h vs. 24 ± 21 ppm·14 h), likely due to its lower absorption efficiency in the small intestine, allowing more to reach the colon.

Our study accounted for roughly half of the ingested 5 g AM (~30% excreted in urine and 22% fermented). In vivo behavior of the remaining ~48% is an important question. The absorption mechanism of AM appears to be key. We observed a dose-dependent difference in bioavailability: the 1 g dose yielded a higher urinary excretion rate (58% vs. 30%) and lower fermentation rate (6% vs. 22%) compared with the 5 g dose. This pattern strongly suggests a saturable transport process, similar to that reported for fructose [19,20]. At higher doses, this transporter may be saturated, allowing more unabsorbed AM to pass into the large intestine for fermentation. Our complementary rat study offers insight into the missing fraction: fecal excretion of AM was minimal (<1% of the dose). This implies that most of the unaccounted portion was absorbed and underwent a different physiological pathway. For the fraction absorbed but not excreted in urine, a leading hypothesis is renal tubular reabsorption and subsequent retention in the body. This mechanism is known for structurally related compounds; notably, SGLT5 is the primary renal transporter for 1,5-anhydroglucitol and is strongly competed by AM (1,5-anhydro-D-mannitol), suggesting SGLT5 as the likely mediator of AM reabsorption [21,22]. Joseph et al. reported no significant sex differences in the relative mRNA expression levels of renal transporters, including SGLT5 (SLC5A10), after statistical adjustments [23]. Consistent with this finding, despite the uneven male-to-female ratio in our study, the urinary AM excretion rate was 29.5% ± 6.6% in the overall cohort (n = 15), 34.9% ± 8.2% in males (n = 3), and 28.2% ± 5.3% in females (n = 12); no significant differences were observed between sexes. Therefore, we infer that sex differences do not affect the urinary excretion rate of AM. Another possibility is that a small fraction of absorbed AM is incorporated into cellular components, such as glycoproteins, similar to fucose and mannose [24,25,26]. Therefore, we speculate that neither pathway substantially contributes to energy production. This speculation aligns with our finding of no change in blood glucose levels.

The strength of this study lies in dual-model approach which is its rigorous crossover design in healthy participants and the complementary use of an animal model to assess fecal excretion. However, due to measurement constraints in human studies (e.g., timing of blood and breath gas collection, urinary sampling), the full gastrointestinal and systemic kinetics of ingested AM remain incompletely characterized. To fully elucidate the detailed systemic kinetics and potential secondary intracellular pathways of the unaccounted fraction, further studies utilizing advanced methodologies, such as the continuous intravenous infusion of 13C-labeled compounds or isotopic tracing, are required in the future.

## 5. Conclusions

AM’s structural deviation from direct metabolic pathways, high urinary excretion rate, and low intestinal fermentability consistently indicate that AM is potentially utilized as a low-energy source in humans. Notably, the excretion of ~30% of the ingested dose into urine strongly supports AM’s potential as a functional ingredient capable of reaching its site of action for UTI prevention more efficiently than conventional D-mannose.

## Figures and Tables

**Table 1:** Baseline characteristics of study participants in healthy adults.

	Men (n = 3)	Women (n = 12)
Age (year)	23.0 ± 1.4	21.5 ± 1.8
Height (cm)	168.3 ± 1.7	158.3 ± 4.2
Weight (kg)	61.7 ± 4.6	52.3 ± 5.8
BMI (kg/m^2^)	21.8 ± 2.1	20.8 ± 2.0

**Table 2:** Cumulative urinary excretion of AM and mannose after a single oral ingestion in healthy adults.

	Urinary Excretion Rate (%)			
	0–4 h	4–8 h	8–24 h	0–24 h
AM 5 g (n = 15)	18.7 ± 3.7	6.2 ± 2.3	4.6 ± 1.4	29.5 ± 6.6
AM 1 g (n = 6)	34.3 ± 17	14.7 ± 6.4	9.2 ± 2.5	58.3 ± 25.4
D-mannose 5 g (n = 15)	nd	0.1 ± 0.0	-	-

Values are presented as mean ± SD. The percentage of ingested dose was calculated based on the total amount of AM (5 g for n = 15, 1 g for n = 6) and D-mannose (5 g for n = 15) ingested. AM, 1,5-anhydro-D-mannitol; nd, not detected.

## Data Availability

The raw data supporting the findings of this study are subject to a confidentiality agreement with the industry funder and are therefore not publicly available. All summary data generated during this study are included in this published article.

## Abbreviations

The following abbreviations are used in this manuscript:

AM	1,5-anhydro-D-mannitol
AUC	Area under the curve
BMI	Body mass index
FOS	Fructooligosaccharide
GC-MS	Gas chromatography–mass spectrometry
SD	Standard deviation
SGLT	Sodium–glucose cotransporter
TMS	Trimethylsilyl
UPEC	Uropathogenic Escherichia coli
UTI	Urinary tract infection
